# When are you taking us outside? An exploratory study of the integration of the outdoor learning in preschool and primary education in Quebec

**DOI:** 10.3389/fpsyg.2022.955549

**Published:** 2022-10-20

**Authors:** Audrey-Anne Beauchamp, Yannick Lacoste, Célia Kingsbury, Tegwen Gadais

**Affiliations:** ^1^Département des sciences de l’activité physique, Université du Québec à Montréal, Montréal, QC, Canada; ^2^Chaire UNESCO en développement curriculaire (CUDC), Université du Québec à Montréal, Montréal, QC, Canada; ^3^Centre de Recherche Interdisciplinaire sur la Formation et la Profession Enseignante (CRIFPE), Université du Québec à Montréal, Montréal, QC, Canada; ^4^École de Santé Publique de l’Université de Montréal (ESPUM), Montréal, QC, Canada; ^5^Centre de Recherche de l’Institut de Santé Mentale de Montréal (CRIUSMM), Montréal, QC, Canada

**Keywords:** outdoor education, outdoor learning, nature, preschools, primary schools

## Abstract

**Introduction:**

Recent research investigating the benefits of being outdoors and contact with nature in children showed strong associations with children’s health and development. More teachers are choosing to integrate outdoor learning (OL) into their practice in Quebec, but few studies have focused on OL in the school environment, particularly in Canada and more specifically in Quebec, despite the fact that the school context lends itself favorably to this practice.

**Objective:**

The purpose of this study was to portray OL in preschool and primary schools in Québec by identifying three key elements: (1) teachers’ perception of the outdoors, (2) the uses of OL in schools, and (3) teaching strategies and factors that influence teachers’ integration of OL.

**Methodology:**

Semi-structured group interviews (*n* = 4) conducted with 14 teachers and participant observations (*n* = 4) were used for data collection. Inclusion criteria were to be a preschool or primary school teacher, to have taught at least eight sessions of OL in the past year, and to have no connection or contact with the research team prior to the start of the study.

**Results:**

First, the results showed that teachers commonly understood the outdoors as being in the open air, practicing a physical activity, having the presence of nature, providing physical freedom and targeting a pedagogical intention. Second, teachers appeared to incorporate a variety of pedagogical intentions in OL (e.g., environmental awareness, interdisciplinary learning), in a variety of settings (e.g., city parks, woodlands), and with a variety of academic subjects (e.g., French, mathematics) and learning tasks (e.g., walking, nature shelter building). Third, teachers used a wide range of teaching strategies in OL (e.g., flexible planning, well-established routines). Participants also identified multiple factors specific to their setting that appeared to facilitate (e.g., parental support) or limit (e.g., storage of materials) their integration of OL into the school environment.

**Conclusion:**

This study provided a better understanding of the current use of the OL in the Quebec school environment by identifying the common characteristics, limitations and winning strategies of its use in schools. Teachers and schools interested in OL could benefit from the results of this study, particularly those interested in adopting a *Forest School* or *Udeskole* approach.

## Introduction

### The outdoors for children’s health and learning

Benefits associated with outdoor activities in children are now well established in scientific literature (McCormick, 2017; Schneller et al., 2017; Mann et al., 2022). Particularly, being outdoors would provide benefits not only for physical, psychological, and social health, but also for the practice of physical activity, and for the educational success of children (5–17 years). It appears that being outdoors strengthens their immune system, decreases stress experienced in daily life (Kuo et al., 2019), promotes their interpersonal relationships (Seeland et al., 2009; Keniger et al., 2013; Larouche et al., 2016) and makes them happier (Barrera-Hernández et al., 2020). It also helps to promote the practice of physical activity in children (Alvarez-Bueno et al., 2017; Santana et al., 2017; Bølling et al., 2021) while playing a positive role in their academic performance (Kuo et al., 2019). In addition, being outdoors is reportedly positively related to increased perseverance, self-discipline, attention, problem solving, critical thinking, and interest in school (Kuo et al., 2019). To date, we have not found many studies that showed no effect of the outdoors on children. However, Mann et al. (2022) explains that at this point, it is difficult to know whether it is contact with nature or simply teaching methods that have an impact when comparing outdoor versus indoor education.

Despite all the demonstrated benefits, disconnection from nature seems to be an increasing phenomenon among children in recent years (Louv, 2008; Strife and Downey, 2009; Cardinal, 2010; Silverman and Corneau, 2017), more specifically in the school environment (Waite, 2010) and several studies reveal that actions need to be taken to address this issue (Chawla, 2015; Soga and Gaston, 2016; Kahn and Weiss, 2017). In this respect, several studies indicate that the school setting appears to be an ideal context for encouraging outdoor activities among youth (Hills et al., 2015; Bentsen et al., 2021). Thus, integrating the outdoors in education appears to be part of a complementary health promotion strategy that would allow children to benefit from all of these effects (Nielsen et al., 2016).

### The lack of research on outdoor education in Quebec

In Quebec, there is a growing interest in integrating the outdoors into the school environment (Maziade et al., 2018; Gadais et al., 2021a; Ayotte-Beaudet et al., 2022). To this end, results from a survey conducted by the Fondation Monique-Fitz-Back (2018) indicate that 75% of school-based practitioners conduct educational projects in outdoor settings in Quebec schools. In addition, in 2017, the Ministry of Education published a scientific report promoting the inclusion of outdoor activities in the school program (Ministère de l’Éducation et de l’Enseignement supérieur du Québec, 2017) and now indirectly encourages it through initiatives such as *15,023 À l’école, on bouge au cube*! Despite the government’s efforts and the perceived excitement, Quebec teachers still face many challenges in integrating the outdoors in preschool and primary school (Ayotte-Beaudet et al., 2022).

The results available to date indicate that there are few studies on the use of the OL by preschool and primary school teachers in Quebec. Indeed, scientific literature reveals little information regarding preschool and primary teachers’ perceptions of OL and there appears to be no scientific consensus regarding the definition of the outdoors (Gadais et al., 2021b). In addition, studies that focus on the organization of current outdoors initiatives, as well as on the description of effective outdoor pedagogies, appear to be lacking (Ayotte-Beaudet et al., 2017). Only a few Quebec studies have identified factors that limit or facilitate (Maziade et al., 2018; Sport et loisir de l'Île de Montréal, 2019; Ayotte-Beaudet et al., 2022) OL in preschool and primary schools. Finally, there is a lack of available didactic tools and existing pedagogical approaches to support teachers in their integration of OL in their practice (Maziade et al., 2018). Therefore, it seems relevant to study existing OL practices in preschool and primary schools in order to better understand the characteristics that determine OL in Quebec and to offer effective and accessible theoretical anchors to teachers who wish to use it.

The choice to study OL in preschool and primary schools (4 to 12 years) is based on several factors. First, preschools and primary schools act as a favorable environment for the adoption of healthy lifestyle habits. During this period of time, lifestyle habits are formed and can have a positive long-term influence, such as maintaining physical activity practice until adulthood (Janz et al., 2005). Second, it is also during this period of their lives that children derive maximum benefits from contact with nature (Moens et al., 2019), that can have lifelong repercussions (Townsend et al., 2015; WHO, 2016). Third, positive experiences with nature during childhood could promote continued engagement with nature and the promotion of pro-environmental attitudes (Sachs et al., 2020).

### Foundations and pedagogical approaches of outdoor learning

**Figure 1:**
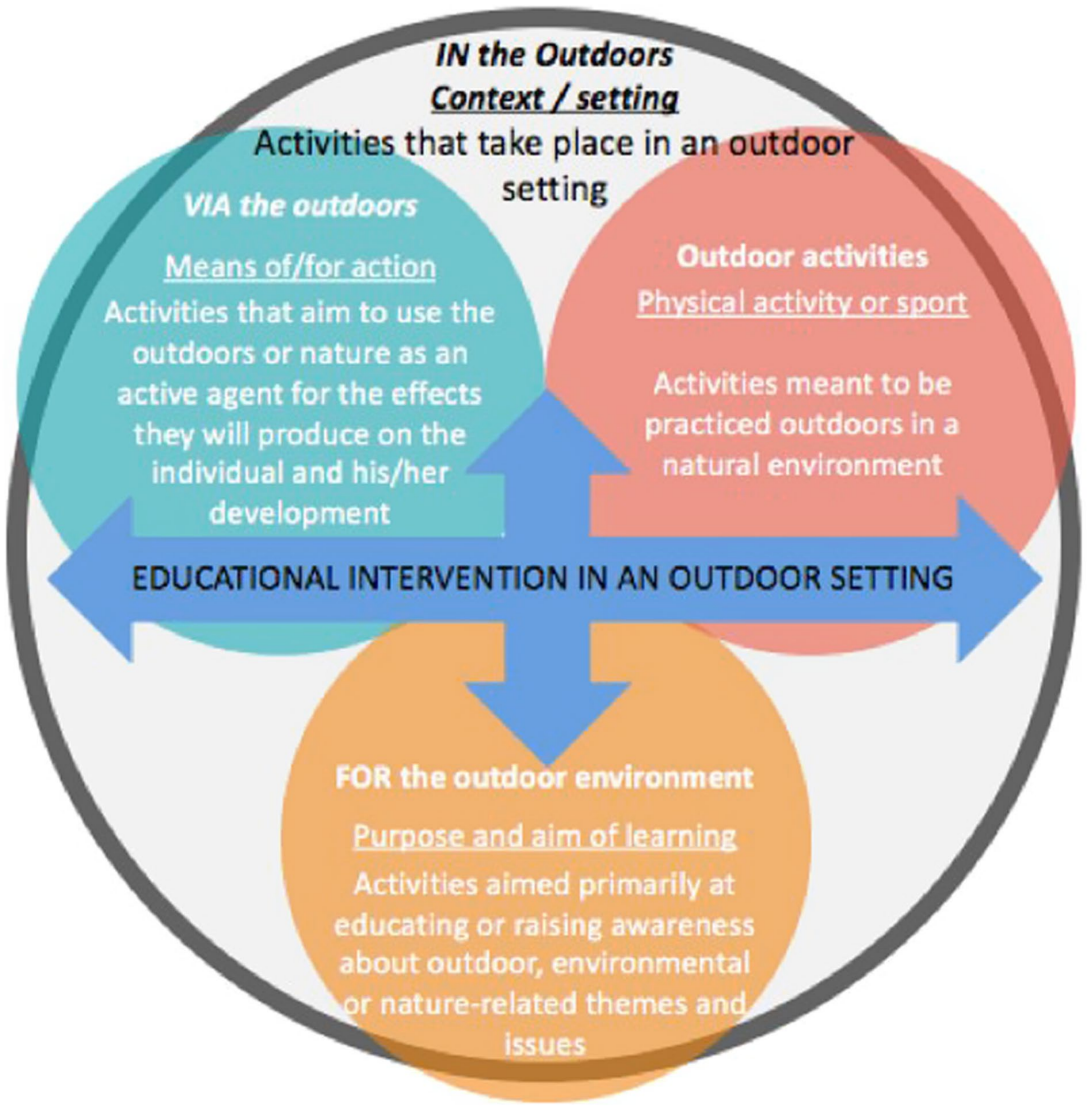
Intentions to use the outdoors by Gadais et al. (2021b).

OL is a broad field that employs a variety of approaches depending on regions and cultures (Gadais et al., 2021a). In this study, we drew on different approaches to better situate current teaching practices in relation to one another and through the conceptual framework of the Educational Intervention Model in the Context of outdoors (IECPA – *intervention éducative en contexte de plein air*) (Gadais et al., 2021a). This model, also referred to as the intentions to use the outdoors matrix, is designed to conceptualize the various intentions and contexts of the outdoor use. This model aims to define the variety of outdoor activities (e.g., orienteering, bicycle), and activity practices *in* the outdoors (e.g., *Udeskole*) *via* the outdoors (e.g., *Adventure Education, Forest School*), and *for* the outdoor environment (e.g., *Environmental Education*). In painting a picture of the integration of OL by preschool and primary school teachers, it was possible to identify the conditions favorable to expanding the implementation of OL and to put forward recommendations to promote its development in the Quebec school environment. Figure 1 presents the intentions to use the outdoors matrix.

Thus, we use the term *Environmental Education* to refer to a stream of thought and action that aims primarily to promote the emergence of eco-citizens by responding to environmental, educational and pedagogical issues (Sauvé, 2015). In the Anglo-Saxon culture, we find *Adventure Education*, which is an experiential type of educational approach that immerses the participant in a sense of uncertainty or insecurity in order to encourage them to surpass themselves and achieve personal development (Sibthorp, 2003; Priest and Gass, 2018). *Forest school*, also referred to as nature-based pedagogy, uses nature as a learning environment and vehicle to provide children with socioconstructivist and inclusive learning experiences (Maynard, 2007; Coates and Pimlott-Wilson, 2019). Finally, in Scandinavia, there is *Udeskole*, which translates to school outside, by focusing on mandatory and regular educational activities outside the school walls (Bentsen and Jensen, 2012). Other streams and approaches to OL exist, but those selected for this study as the most likely to support the three research objectives. Table 1 provides a synthesis of these four known approaches in OL.

**Table 1:** Synthesis of outdoor education approaches.

Approaches	Concepts	Main effects
*Adventure education (AE)*	A form of experiential education that focuses on the development of the person by immersing them in a sense of uncertainty or insecurity in order to challenge them (Sibthorp, 2003; Priest and Gass, 2018).	Decrease mental stress, promote self-efficacy, mindfulness and well-being, strengthen group cohesion and individual responsibility towards others, help solve problems such as truancy and depression (Harper, 2017).
*Environmental education (EE)*	Aims primarily to foster the emergence of eco-citizens who live a conscious, creative and committed citizenship by addressing environmental, educational and pedagogical issues (Sauvé, 2015).	Promotes a critical approach (Sauvé, 1997); Allows the development of environmental knowledge, will and power to act (Sauvé, 2015).
*Forest school (FS)*	Aims for children to spend the majority of their days in nature, often in the forest and in a variety of weather conditions by encouraging learning through free play, motor skills development, exploration of nature, collaboration among learners, and risk taking (Elliot et al., 2014; Coates and Pimlott-Wilson, 2019).	Improved creative, problem-solving, self-directed and collaborative learning skills, increased physical activity practice, creation of stronger social support networks, recognition of personal, social and environmental responsibilities, development of resilience, development of social skills such as conflict management, negotiation and diplomacy (Coates and Pimlott-Wilson, 2019);
*Udeskole*	Characterized by mandatory and regular educational activities outside of school buildings, especially in natural and cultural settings (e.g., forests, parks, local communities, factories and farms) (Bentsen and Jensen, 2012).	Optimize students’ physical activity practice; Promote pro-social behaviors (Bølling et al., 2019); Increase academic motivation (Bølling et al., 2018); have a positive effect on social behaviors, attitudes toward teaching and toward learning and physical activity practice (Mygind, 2009)

### Current research on teaching strategies

**Figure 2:**
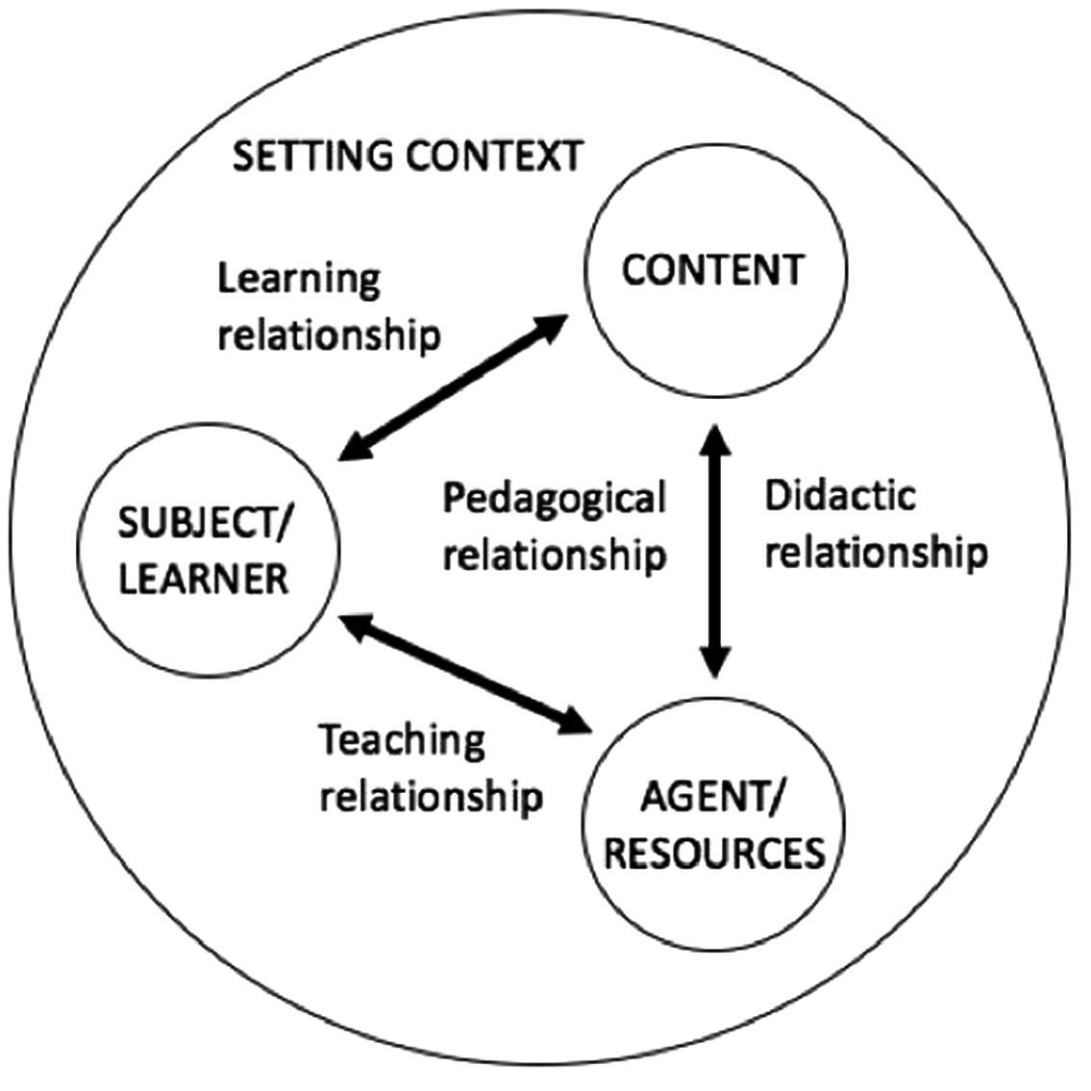
SOMA model (Legendre, 2005) (p.1240).

The teaching strategies (pedagogical and didactic strategies) presented in the following section are directly derived from Legendre’s SOMA model (Legendre, 2005), which summarizes the pedagogical and didactic situation in education (Figure 2). This model aims to shed light on the educational relationships that exist between the three poles of the pedagogical relationship in education, i.e., between the agent or the resources (teacher), the subject or the learner (student) and the content (e.g., knowledge, learning), with the goal of supporting the development of the individual while considering his or her needs and the setting context. The didactic relationship (between the teacher and the knowledge) and the teaching relationship (between the teacher and the student) are investigated in this study as teaching strategies. They are defined as any intervention that is used to support learning. The scientific gap on these strategies used in OL prompts us to study those that seem the most relevant to OL and to explore them further in relation to the objectives of this study.

The teaching strategies studied include, first, the pedagogical relationship, which represents all the exchanges, reciprocal influences, actions and reactions between the teacher and the student (Weigand and Hess, 2007). As a fundamental condition for educational effectiveness (Cosmopoulos, 1999), studying the pedagogical relationship in OL will allow a better understanding of teachers’ practices in OL. Secondly, there is group management, which is the set of educational practices that the teacher puts in place to allow optimal teaching and learning conditions (Doyle, 1986). According to a recent Quebec study (Ayotte-Beaudet et al., 2022), student management is one of the avenues to be explored further in OL. Third, teacher planning is based on the perception of students’ needs (Tochon, 1993), which is used in education to organize teaching-learning content (Yinger and Clark, 1982, 1983). It seems relevant to study the planning methods used by teachers in order to better understand how they can support OL. Finally, the environment, which is a central space that can represents the setting context in which teaching and learning take place (Legendre, 2005). Focusing on the environments used by teachers in OL will provide a better understanding of their characteristics and uses. Table 2 presents a synthesis of the teaching strategies used in this study and the associated concepts.

**Table 2:** Synthesis of the teaching strategies (pedagogical and didactic) used in this study and associated concepts.

Strategies		Associated concepts
Pedagogical	Pedagogical relationship	Exchanges, reciprocal influences, actions and reactions between the teacher and the student (Weigand and Hess, 2007);
		Benevolence and empathy (Visioli, 2019).
	Group management	All the educational practices put in place to allow teaching and learning conditions (Doyle, 1986); Optimal internalization of the rules (Méard and Bertone, 2009).
	Environment	The outdoors as an authentic educational context (Ayotte-Beaudet et al., 2020); Three levels of use: inspiration, pedagogical tool, interdisciplinary learning (Moffet, 2019).
Didactic	Planning	Juxtaposition of content related to students’ perceived needs (Tochon, 1993); Simplifies and organizes teaching-learning (Yinger and Clark, 1982, 1983); Place of predictability and unpredictability (Tochon, 1993); Routines (Yinger, 1979).

### Limiting factors of outdoor learning integration

Several factors influencing the integration of OL into the teaching environment have been unanimously identified in the literature. These include the lack of teacher confidence and expertise (Higgins et al., 2006; Barfod, 2018; Edwards-Jones et al., 2018; Van Dijk-Wesselius et al., 2020), the lack of time to prepare and conduct activities (Edwards-Jones et al., 2018; Van Dijk-Wesselius et al., 2020), the lack of access to outdoor sites (Higgins et al., 2006; Waite, 2010), the lack of funding (Waite, 2010) the lack of support for OL (Ruether, 2018), the difficulty to get started (Van Dijk-Wesselius et al., 2020), the physical constraints (Van Dijk-Wesselius et al., 2020) and weather conditions (Ruether, 2018). To date and in Quebec specifically, studies support these observations (Maziade et al., 2018; Sport et loisir de l'Île de Montréal, 2019).

### Objectives of the present study

The purpose of this study is to provide a portrait of the integration of OL in preschool and primary school settings by answering the following question: What are the teaching strategies (conceptions, uses, teaching strategies and influencing factors) that preschool and primary school teachers in Quebec use to integrate OL into their practice? More specifically, the objectives are to: (1) collect and characterize preschool and primary school teachers’ perception of the outdoors, (2) list the uses of OL by preschool and primary school teachers; (3) identify the teaching strategies and factors that influence OL by preschool and primary school teachers.

## Methodology

### Research design

This study used an exploratory qualitative design to meet the three research objectives. A qualitative approach is used in this study since it allows the identification of the reality of the practices and the specific needs of the target population (Dano et al., 2004). It seeks to produce new knowledge on OL in the preschool and primary school setting, a field that has been just little studied in Quebec (Trudel et al., 2006). Data were collected through group interviews (*n* = 4), from groups of three to five teachers (*n* = 14), and through participant observations with several teachers (*n* = 4). A triangulation of the data (Van der Maren, 1996) was then carried out using two types of data: the field notes from the logbook and the data from the group interviews. A cross-tabulation of the data was finally carried out in order to address the three research objectives.

### Recruitment of participants

To be included in the study, participants had to meet the following inclusion criteria: (1) to be a preschool or primary school teacher in the province of Quebec, (2) to have use OL for at least eight sessions in the last school year, and (3) to have no connection or contact with the research team prior to the start of the study. First, teachers were recruited *via* the Internet, through the dissemination of a message to the mailing lists of the Federation of Physical Educators Teachers of Quebec (FEEPEQ – *Fédération des éducateurs et éducatrices physiques enseignants du Québec*), the Quebec School Services Center (*Centre de services scolaires du Québec*), and through a network of contacts in the OL community to re-distribute the message by e-mail. They were automatically selected if they met the inclusion criteria and were available to participate in the study. Then, in a second phase, four teachers were selected for an observation session. A total of 14 preschool and primary teachers were recruited to provide a comprehensive picture and to achieve data saturation (Gainer, 1995). At the primary level, five health and physical education teachers and three classroom teachers were recruited, while at the preschool level, six classroom teachers were recruited for this study. Ten of the teachers worked at Quebec School Services Center schools and one in a private sector school. In total, nine women and five men teachers were recruited. Years of experience in OL of the participating teachers ranged from 0–5 years (7 teachers), 5–10 years (3 teachers), 10–15 years (1 teacher), 15–20 years (2 teachers), and over 20 years (1 teacher). Most participants had an average of 0–10 years of teaching experience in OL. Participants socio-demographic characteristics are presented in Table 3.

**Table 3:** Profile and characteristics of participating teachers.

ID	Sex	Teaching area	Level	School service center	Experience in OL (years)	Frequency of use
P_1	M	Health and physical education	Primary	Marguerite-Bourgeoys	15–20	+
P_ 2	M	Health and physical education	Primary	des Affluents	0–5	+/−
P_3	M	Health and physical education	Primary	Kamouraska Rivière-du-Loup	15–20	+/−
P_4	M	Health and physical education	Primary	des Samares	20+	+
P_5	F	Class teacher	Primary	des Affluents	5–10	+/−
P_6	M	Health and physical education	Primary	des Trois-Lacs	10–15	+/−
P_7	F	Class teacher	Primary	Marguerite-Bourgeoys	5–10	+
P_8	F	Class teacher	Preschool	des Hauts-Bois-de-l’Outaouais	0–5	++
P_9	F	Class teacher	Preschool	des Appalaches	0–5	++
P_10	F	Class teacher	Preschool	de la Rivières-du-Nord	0–5	++
P_11	F	Class teacher	Preschool	des Affluents	0–5	+/−
P_12	F	Class teacher	Preschool	des Navigateurs	0–5	N.A.
P_13	F	Class teacher	Primary	Private	5–10	++
P_14	F	Class teacher	Preschool	des Rives-du-Saguenay	0–5	++

The four colors are used to represent the four interviews conducted with the participating teachers. For example, blue for interview 1 with P_1 to P_5.

++: every day; +: several times a week; +/−: once a week; −: a few times a year

### Procedure

First, four semi-structured group interviews were conducted with the teachers. The average length of the interviews waś approximately 90 min in order to obtain meaningful data (Dano et al., 2004). A total of 14 teachers were interviewed, in subgroups of three or five preschool and primary teachers. An audio recorder was used and the transcripts were done manually and confidentially in verbatim form. Group interviews were used as a data collection tool to avoid subjecting teachers to the principal investigator’s questions alone, to open up dialog and to welcome emergent data from participants (Morrissette, 2022). The interviews was divided into three categories, which represent the three objectives: (1) the perception of the outdoors, (2) the use of the outdoors and (3) the teaching strategies and the factors that influence OL (see Supplementary file for the full version of the group interview guide).

Second, observations of participants (Paré, 2014; Chevalier et al., 2018) lasting between 60 and 180 min were conducted with four teachers who also participated in group interviews. These participant observations allowed for full immersion in the teachers’ practices in OL. They were used to enrich the answers related to the three research objectives and to confirm the data through concrete observations directly in the field. All three observations took place in urban settings. Observation 1 and 2 took place with 10–11 years old students, but observation 1 was in a forest away from the school, while observation 2 took place in a municipal park near the school. Observations 3 and 4 were both conducted with 5–6 years old students, with observation 3 being on the schoolyard and observation 4 being in a forest away from the school. These observations were collected by taking pictures and making quick notes using key words in a handwritten logbook (Paré, 2014). These notes were then analyzed, as were the transcripts of the group interviews. Table 4 presents the data collection process according to the two instruments used.

**Table 4:** Data collection process.

Months	Data collection							
	Group interviews				Participant observations			
	Interview 1	Interview 2	Interview 3	Interview 4	Observation 1	Observation 2	Observation 3	Observation 4
November 2021								
December 2021								

The months of the year when the measuring instruments were used for data collection are in gray.

### Data analysis

Following the data collection, the qualitative data from the group interviews (verbatim) and participant observations (logbook) were transcribed and analyzed using NVivo 12.6 Software. For the analysis, the deductive grid was constructed prior to data collection based on the three research objectives. The data were analyzed using content analysis (L'Écuyer, 2011) in three steps: (1) preliminary reading and listing of statements, (2) selection and definition of classification units, and (3) categorization process. First, the verbatim and the logbook were read twice by the principal investigator to become familiar with the content. This step allowed the principal investigator to obtain an overall picture of the information and identify key trends. Second, the principal investigator proceeded to identify the meaning units, which represented the categories used to address the research objectives. In this step, the principal investigator focused on the information present in the verbatim and grouped it into categories according to the defined objectives and certain emerging categories. The fidelity of this step was ensured by consensus validation between the principal investigator and a member of the research team to make the choice of statements. Third, each unit of meaning was coded and classified into broad categories and specific subcategories. This was an open-ended and semi-inductive categorization (based primarily on the categories in the interview guide). The coding was validated by a member of the research team, through a process of confrontation of the interpretations and reaching a consensus for further analysis. This categorization allowed for the emergence of definitive themes and categories, while drawing on the initial categories of the interview guide and the logbook. During this process, a consensus was reached around four emerging categories and their subcategories (Stake, 1995).

### Ethical considerations

An ethics certificate (2022–4,152) was obtained from the Research Ethics Committee for Student Projects (CERPE plurifacultaire – *Comité d’éthique de la recherché pour les projets étudiants*) of Université du Québec à Montréal. Written informed consent to participate in this study was provided by the participants. Written consent was also provided for the photo taking by the teacher who was observed and by the parents of the students observed.

The ethics committee waived the requirement of written informed consent for participation. Written informed consent to participate in this study was provided by the participants. Written consent was also provided for the photo taking by the teacher who was observed and by the parents of the students observed.

## Results

**Figure 3:**
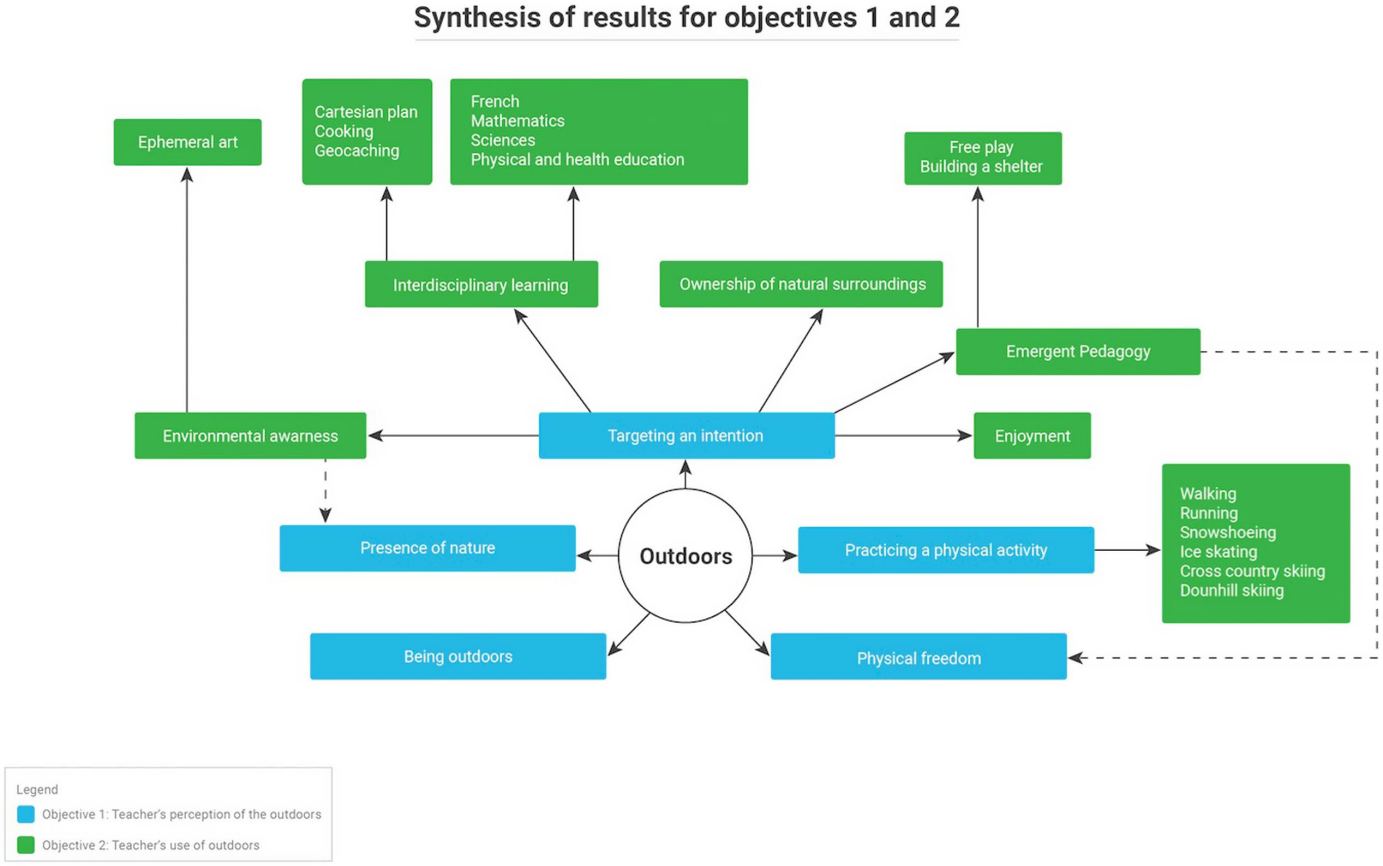
Synthesis of results for objectives 1 and 2.

**Figure 4:**
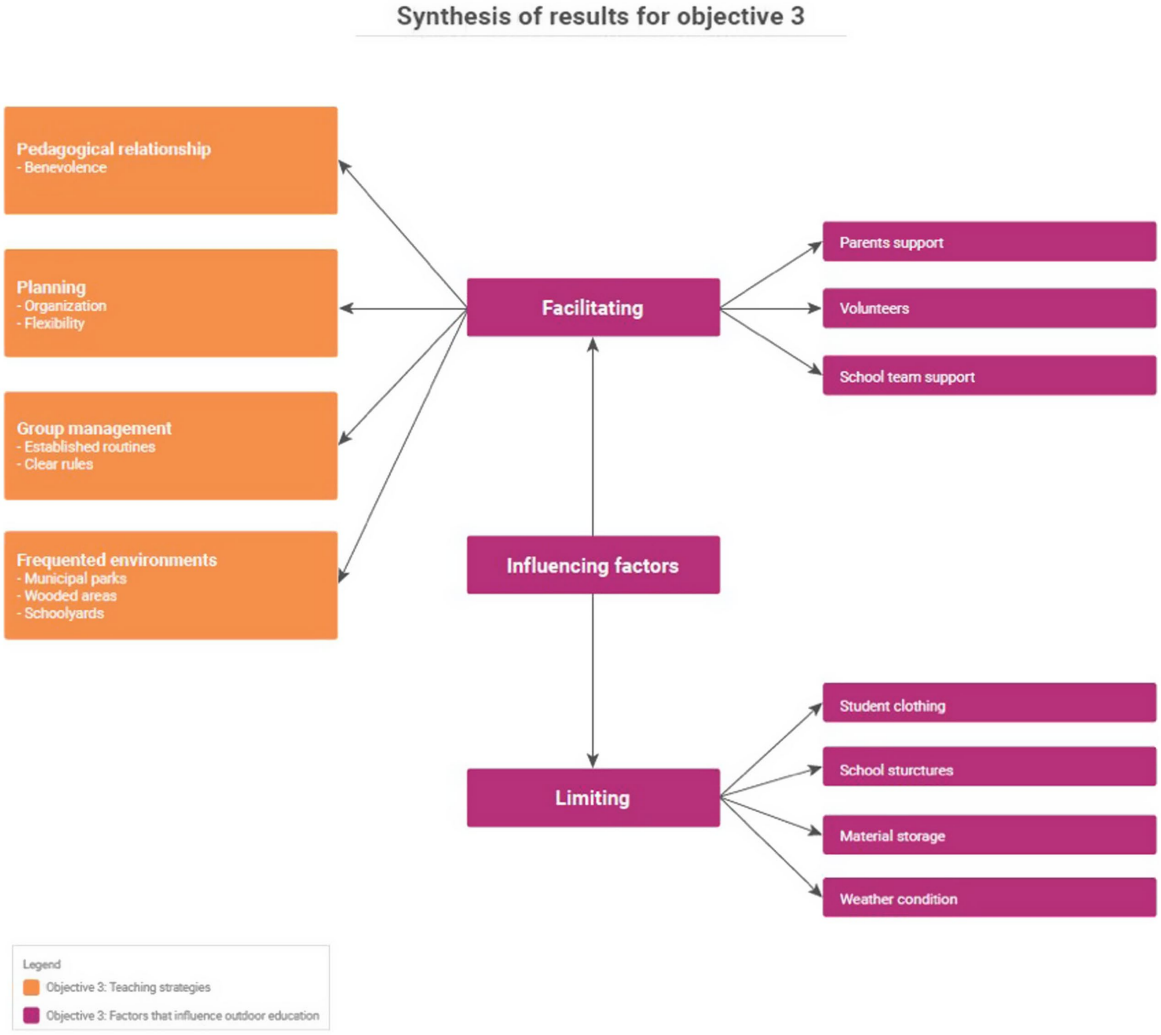
Synthesis of results for objective 3.

The results from the verbatim and logbook records are organized into three main sections to echo the objectives of this research: (1) teachers’ perception of the outdoors, (2) teachers’ uses of OL, and (3) teaching strategies and factors that influence OL. A synthesis of the findings is presented in Figures 3, 4.

### Teachers’ perception of the outdoors

In order to address the first objective of this research, data were collected regarding teachers’ perception of the outdoors. In general, all of the teachers interviewed and observed approached the outdoors from fairly similar perspectives. Five main elements emerged: (1) being outdoors, (2) having the presence of nature, (3) practicing a physical activity, (4) providing physical freedom, and (5) targeting a pedagogical intention.

#### Being outdoors

Teachers’ perception of the outdoors was almost unanimous about being outdoors (*n* = 13/14), as most of them mentioned that the outdoors was associated with being in the open air, being outside, or outside the walls. Nearby, remote, or biodiverse environments also seemed to be perceived as outdoors by the majority of the teachers.

"(...) outdoors, it's really just outdoors. But we can be outside in the schoolyard, we can be outside at the park next door or go to the great outdoors further away." (P_1)

#### Having the presence of nature

Although most teachers felt that outdoors could be done in any setting outside the walls, the presence of nature also seemed to be important in their perception of outdoors, as several (*n* = 5/14) named it.

"Outdoors means (...) being outside, but maybe not outside in a mall parking lot. There's the nature, environment aspect too in the word outdoors." (P_5)

However, one teacher emphasized the possibility of bringing outdoors indoors, by bringing nature in.

"I would even add, you know, it's not just outside. You know, nature can be brought inside in all kinds of ways." (P_9)

Thus, outdoors seems to be associated with elements of nature or the outdoor environment by teachers.

#### Practicing a physical activity

Several teachers (*n* = 4/14) associated outdoors with being physically active or being in motion.

"Q: I would like to know now what the term outdoors means to you? We can finish the interview well with this.

(...)

P_12: Moving (...). I would go with that.

#### Providing physical freedom

Also, several teachers (*n* = 4/14) associated outdoors with the physical freedom it provides to the students while the learning sessions.

"I think it's freedom, it's really freedom. You know, in the classroom, we're there all the time, don't make too much noise, there are classes next door. Now, it's like hey, it's really the freedom aspect where I find that the kids are always being asked to stop talking, to sit down, to get in line, to get back in line, to get back in line again it’s recreation, it’s recess, to get back in line again it’s physical education, to get back in line, get back in line... But now, there's no line." (P_13)

#### Targeting a pedagogical intention

Finally, a few teachers (*n* = 3/14) mentioned that a pedagogical intention was needed to guide the activities in OL, as they need to generate learning to be considered in OL.

“I think there has to be an intention, whatever the intention is. If you go outside and do not do anything.... (...). So, your intention has to be to go listen to the birds, that’s okay. You have an intention, you have a goal in mind (P_7).

### Teachers’ uses of outdoor learning

In order to address the second research objective, data on teachers’ uses of OL were collected in order to inventory them. These include a variety of intentions, school subjects, learning tasks, frequented environments and material used.

#### Pedagogical intentions

The pedagogical intentions used by teachers consisted of the intended goals of using OL and were expressed through the results of this study in five forms: (1) ownership of the natural surroundings, (2) environmental awareness, (3) emergent pedagogy, (4) enjoyment, and (5) interdisciplinary learning.

##### Ownership of natural surroundings

Several teachers (*n* = 6/14) mentioned that they wanted to encourage students to take ownership of the natural surroundings they were visiting, whether it was to make them aware of the environment around them or to encourage them to return to these environments outside the school context.

"Basically, one of the goals of the outdoor program, which I did not mention earlier, is to help students discover the neighborhood. The school, there's a super beautiful city. There are really extraordinary places that the children do not use, parks that they do not know. The point of that is to really discover the entire environment around them." (P_6)

Few teachers (*n* = 3) also wanted students to develop a sense of place or neighborhood, so they could learn about their surroundings.

"There are really several spaces. There's the church, there are markets, the convenience store, so we go there too. We went to get a pumpkin recently. We went by bike, we went to the market to get a pumpkin. This week, we're going to ride our bikes to the post office to mail a letter to Santa Claus. That's what's fun about the village, we're still close. Last year we went to the municipality to get trees. Then, we went to plant them in the forest. There is a greenhouse that gave us soil, we have a sponsorship. We have a hardware store not far away that we are able to go to get materials, we have a painting project right now. So we're really using the environment." (P_14)

##### Environmental awareness

Several teachers (*n* = 6/14) appeared to be doing activities that aimed at having a connection with the environment, develop a greater sensitivity to the environment, or have a better understanding of the environment around the students. When this intention is used, nature seems to be the very object of learning.

"There is a very environmental side that I want to develop in children. So how do we protect things that we don't know about? You take care of what you know and then what you love. So if they have a link with nature, they will know how nature works. We're going to know that our actions have a consequence, since we're all interrelated, that humans are also animals." (P_10)

##### Emergent pedagogy

On the other hand, emergent pedagogy seems to be part of the pedagogical intentions used in OL by several teachers (*n* = 5/14).

"(...) I couldn't tell you, I'm doing this or that because it's really... I'm starting from the children's interests, so it's really emergent pedagogy, so I'm going to do a lot of things." (P_10)

The logbook also corroborated this intention, with key words such as “emergent learning,” “discoveries,” and “exploration” (observation 4).

##### Enjoyment

Pleasure (*n* = 5/14) or enjoyment was a preferred pedagogical intention of several of the teachers interviewed and observed.

"I try as much as possible to have fun all the time. My classes aren't always great, but what I mean is that they need to have fun outdoors." (P_1)

The logbook corroborated this with terms such as “intentions: free play, fun” for observation 3.

##### Interdisciplinary learning

Interdisciplinarity (*n* = 2/14 and one participant observation) was also an intention advocated by few teachers during OL activities.

"I have the classroom teacher who walks us through this. We try to do almost every project. We try to do interdisciplinarity. We do mapping, we work on a cartesian plane, we go on our snowshoeing trip, the kids have to look for the animals, they have to give an oral presentation on the animals, they had the iPad, they film themselves. After that, the teacher makes an assessment outline for an oral presentation. So, we really... we try in almost all our activities to integrate interdisciplinarity. Like recipes today, we are working on proportions, fractions, so we work... we work with all that too." (P_6)

Notes from the logbook corroborated the use of interdisciplinarity as a pedagogical intention, through key words such as “geocaching,” “French,” and “math” in the same session (observation 2).

#### School subjects and learning tasks used

**Figure 5:**
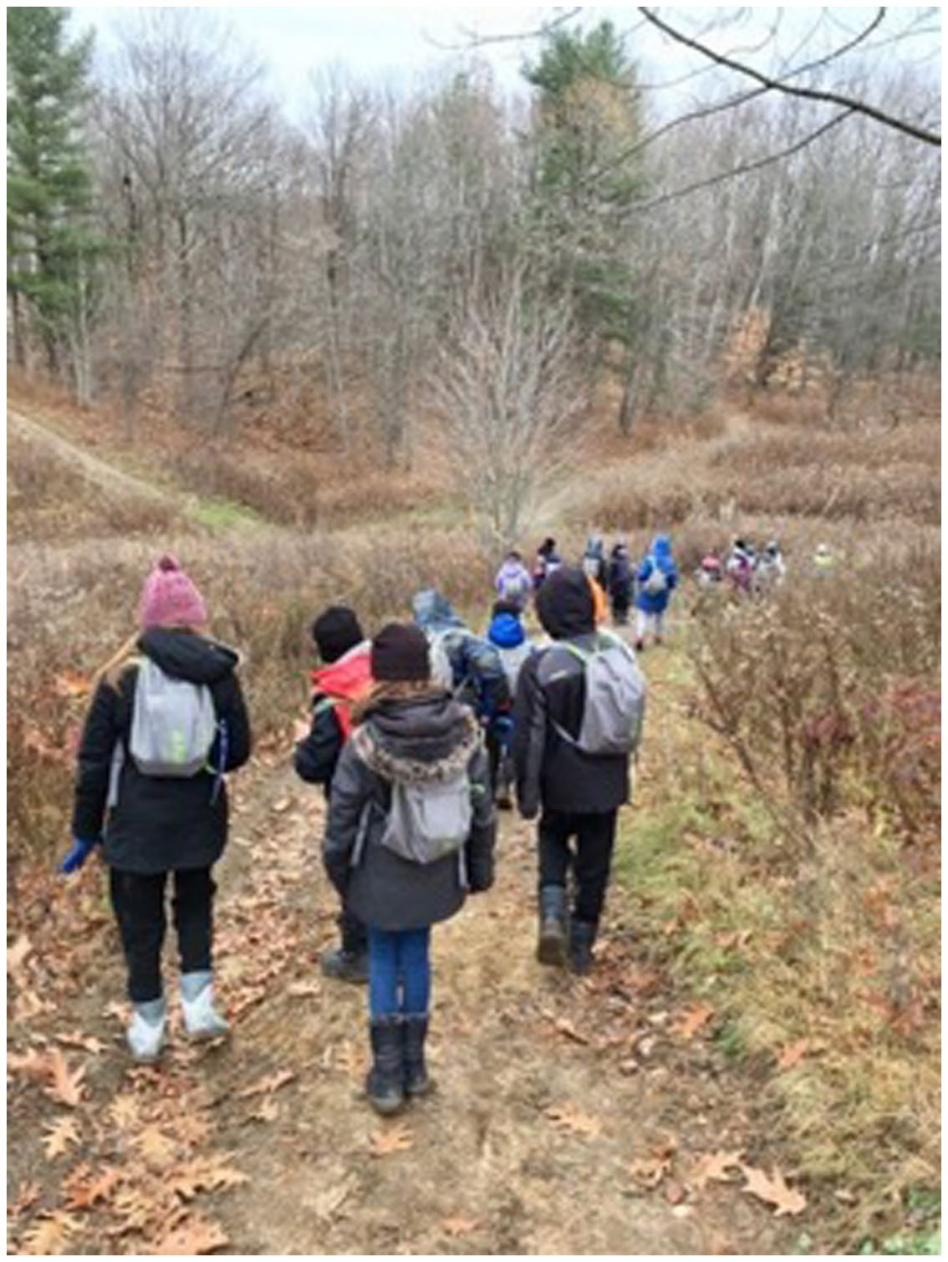
Outdoor learning on foot in a wooded area.

This study identified different ways to teach in OL. These can be divided into (1) school subjects and (2) learning tasks, which include both the *moyens d’action* (Méard and Bertone, 2009) and the learning tasks (Durand and Durand, 2001). The most often taught school disciplines in OL by teachers were French, more specifically writing (*n* = 8/14) and reading (*n* = 5/14), mathematics (*n* = 8/14) and science (*n* = 4/14). Physical and health education (*n* = 5/14) is also taught by the physical and health education teachers specialists who participated in this study. The most common learning activities used in OL by all teachers were walking (*n* = 7/14), free play (*n* = 6/14), nature shelter building (*n* = 6/14), ephemeral art (*n* = 4/14), running (*n* = 4/14) and cooking (*n* = 3/14). Several (*n* = 4/14) also mention starting fires with their students, in the forest or on the school grounds, on which they cook. Figure 5 shows students in the fourth participant observation session participating in a shelter construction in the forest as part of an outdoor class.

"I do all kinds of things. It can be art, math, art, science, reading, writing. Really, anything you can do as a subject in primary school." (P_7)

"We're going to set up the native tent with the little wood stove inside otherwise, it's also fires outside in the forest. So everything to do with cooking outside. We're going to cook everything that's native, we're going to make bannock bread. (P_9)

The logbook corroborated these school subjects and learning tasks, with key words such as “math” and “walking” (observation 1), “science” and “free play” (observation 3), and “shelter building” and “free play” (observation 4).

One preschool teacher also mentioned doing a play-fighting activity, to keep up with the children’s needs for more physical play. This could be confirmed using the logbook, which included the keywords “supervised bickering” (observation 4).

"I know it's in the child's development to play-fighting, but here I do it in a supervised way. I ask them to come to me, and then I turn them around to a place where there are no opportunities, there are no obstacles or trees or rocks nearby. Then there I tell them my safety instructions." (P_10)

In addition, most teachers (*n* = 8/14), including several health and education teachers (*n* = 5/8), engaged in physical activities in OL, such as snowshoeing (*n* = 7/14), ice skating (*n* = 4/14), downhill skiing (*n* = 3/14), and cross-country skiing (*n* = 3/14). One teacher also mentioned doing an introductory camping activity in the schoolyard.

"We do canoeing, running, skating, snowshoeing, introduction to downhill skiing in the schoolyard, we have a little rink in the schoolyard, scootering, biking, Frisbee, as much as possible outside." (P_4)

#### Materials used

In order to provide optimal outdoor teaching, teachers reported bringing a variety of useful items with them. The most common item brought was a first aid kit (*n* = 5/14), followed by a cart (*n* = 4/14), bins or baskets to hold and transport materials (*n* = 4/14), and extra snacks for students (*n* = 3/14).

"Me, I can just quickly add what comes to mind is that every time we go on an outdoor field trip, we leave with walkie-talkie, first aid kit, it's clear, we always have that with us." (P_3)

Few (*n* = 2/14, corroborated by 2 participant observations) also reported bringing a transceiver to ensure communication with the school team, extra clothes for their students, a whistle, and even a saw, nails, screws, and hammers for students.

"We build with saws and hammers and nails. We build animal shelters in the winter." (P_13)

Overall, data from the group interviews indicated that a few teachers (*n* = 3/14) requested that each student have a backpack. The logbook corroborated this with two observations, “every student has a backpack” (observation 1) and “every student has their backpack with their student number” (observation 4).

### Teaching strategies and factors influencing the integration of outdoor education

In order to address the third research objective, data were collected related to teaching strategies and factors that influence the integration of OL into the school environment. First, teaching strategies such as planning, routines, and rules emerged from the group interviews and participant observations and were characterized in OL. Second, factors that facilitate and limit the integration of OL were named by the participating teachers.

#### Teaching strategies

The results of this study allowed us to better characterize the pedagogical and didactic strategies used by the teachers, namely the pedagogical relationship, planning, group management and the frequented environments.

##### Pedagogical relationship

Although the teachers participating in the group interviews did not specifically elaborate on the pedagogical relationship they implement in OL, the four participant observations indicated that it is marked by “closeness between the teacher and the students,” “benevolent pedagogy” (observation 1), “mutual trust” (observation 2), “benevolence” and “calmness” (observation 2 and observation 4).

##### Planning

Teachers expressed that the two greatest strengths of optimal planning in OL are (1) that it is well organized (*n* = 7/14 and 2 observations) and (2) that it is flexible (*n* = 8/14). By organized, teachers meant always thinking about what will be taught ahead of time, preparing materials ahead of time, having a Plan B, and reserving time if needed.

"Yes, you have to be organized, you have to have planned. You have to know where you're going and then you have to organize ahead of time, you can’t be last minute." (P_7)

The logbooks of the four participant observations corroborated organization for optimal planning and session flow through themes such as “organized” and “structured.”

Flexibility or adaptability through planning was also an important element for teachers. They named the possible contingencies and the importance of being able to react and adapt quickly to any eventuality.

"I would say that you have to adapt, you have to be able to adapt as well. It might not go as planned so I think you have to have the ability to adapt quickly. There are times when we do activities that don’t go the way we thought it would." (P_7)

##### Group management

The internalization of rules by students seems to be part of useful pedagogical strategy for optimal group management in OL. Several rules were named by the teachers, but those related to geographical boundaries (*n* = 10/14) and those related to safety (*n* = 7/14) seemed to represent the two main categories of the most used rules.

First, rules related to geographic boundaries often referred to expected student behavior or landmarks that should not be crossed.

"But yeah, otherwise me, what I really like to do is always show them the boundaries before I leave them. No matter what I do when we get there, this is our place, and then these are our boundaries. You can never go beyond these limits, and after that I don't often have to repeat them. (P_11)

The logbook of the second observation corroborated the rules about geographical limits, through the following key words: “pre-established limits (street names).”

Next, teachers indicated that safety instructions refer to what students must follow in order to ensure the safety of all during the activity. For example, not climbing trees, not throwing objects, or not putting anything in your mouth are rules that have been named.

"I don't want them to climb trees, throw... they are very small so the branches, you leave them on the ground. There's no one playing with swords, there's no one throwing rocks at each other, it's really basic rules, safety rules." (P_8)

Two participant observations noted that building on student autonomy seems to be part of an effective group management strategy in OL. The logbook indicated “autonomous students” (observation 1 and observation 2), “emphasis on student accountability and autonomy, teacher does not have much to do” (observation 3), and “free and autonomous students, effective classroom management” (observation 4).

On the other hand, routines also seemed to be part of an effective group management. Several (*n* = 5/14) said that they did the morning routine with the students inside, just before going out. Next, they named different types of routines used in OL education, including the routine for rallying students (*n* = 4/14). To do this, they used various means, such as a song or animal call, to get students’ attention and bring them back to a place.

"Then my routine too is at the wolf howl they come back to the assembly point." (P_10)

To this end, the logbook from observation 1 corroborated the routine for rallying by presenting terms such as “the teacher says 1, 2, 3, LEGO to bring them back.” Observation 4 corroborated the rallying routine with key words such as “wolf howl for gathering and moving.”

Finally, two preschool teachers also associated a routine with a time of connection to nature and the environment, where spiritual values seem to be emphasized.

"There's also a routine of gratitude. We say hello to the sun, we appreciate, thank you. A lot of native values too, we go, we really go but it's like everything is alive. The rock, we become aware of it." (P_10)

##### Frequented environments

**Figure 6:**
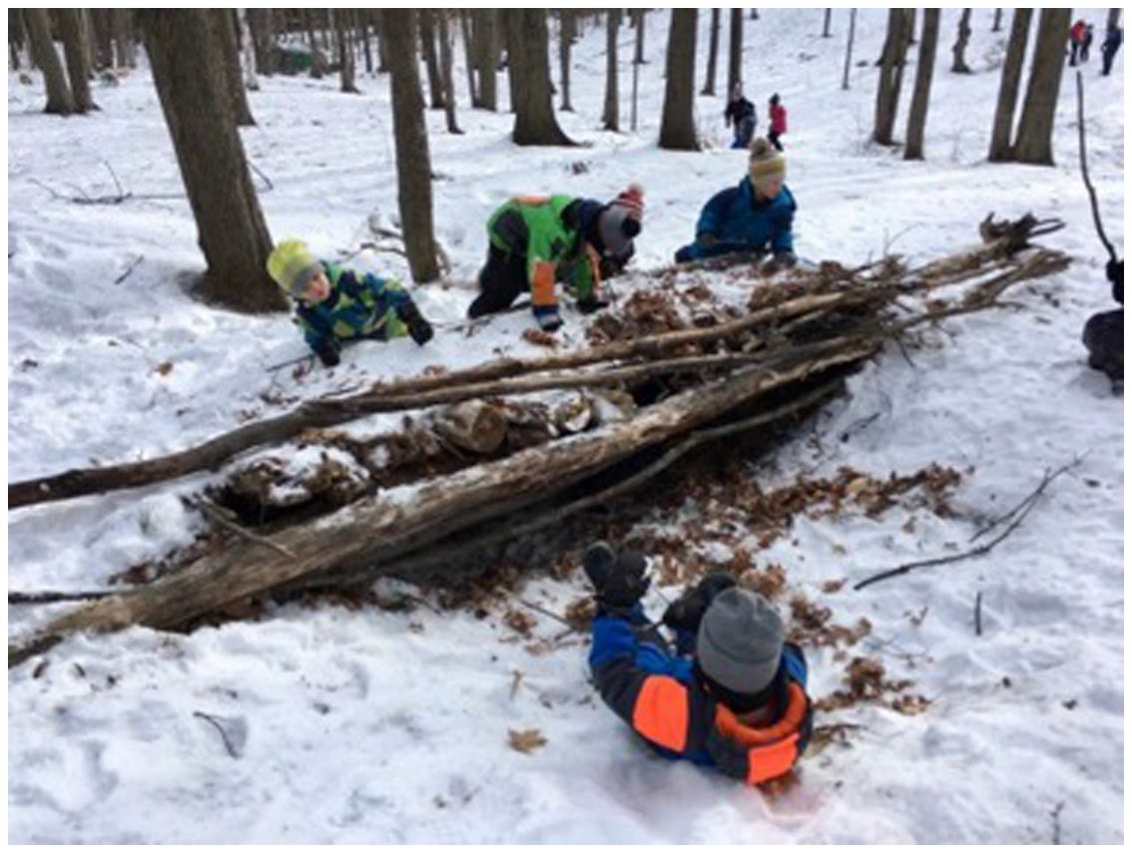
Students build a shelter as part of an outdoor class.

The most frequently visited environments by teachers were municipal parks (*n* = 12), wooded areas (*n* = 12) and schoolyards (*n* = 10). These were environments close to the schools and therefore within walking distance. Figure 6 shows students participating in an educational walk in the woods as part of an OL course observed during the first participant observation session.

The logbook corroborated this data with some key words. For use of city parks, we note “use of park within five-minute walk” (observation 2). For use of wooded areas, we note “walking in the forest” (observation 1) and “use of the forest behind the library” (observation 4). For schoolyard use, “use schoolyard” (observation 1) and “use park in schoolyard” (observation 3).

Skating rinks (*n* = 7/14) and bodies of water (river, lake, or stream) (*n* = 6/14) are also used by several teachers interviewed. It is interesting to note that churches (*n* = 2/14), libraries (*n* = 1/14), cemeteries (*n* = 1/14), businesses such as markets, convenience stores or hardware stores (*n* = 1/14), post offices (*n* = 1/14) and municipal offices (*n* = 1/14) were also environments frequented in OL by few study participants.

#### Factors that influence outdoor education

Factors that influence the integration of OL in the school setting were categorized into two perspectives: factors that help or facilitate the integration of OL and factors that limit or hinder the integration of OL.

##### Facilitating factors

The factors most mentioned as helping teachers integrate OL into their practice were the support of parents (*n* = 10/14), the presence of volunteers (*n* = 9/14) and the support of the school team (*n* = 9/14).

"Simply put, it's school-family-community. If you have those three opportunities with you, it’ll go well and you'll have resources and then you’ll be able to do all these ideas if those three spectrums work with you." (P_4)

The logbook also reports the presence of volunteers, with these key words: “very supportive parent-volunteers” (observation 2).

Several teachers (*n* = 6/14) also mentioned having material or financial support and a few (*n* = 4/14) mentioned being paired with a colleague or having a schedule that allows them time to teach OL. Finally, fun was also mentioned as an important, even helpful, element in OL (*n* = 3/14).

"The challenge is to equip them to want to go by themselves. That they realize that it is fun and that yes, we have fun as P_1 said. It's a goal that you have to keep, but you have to have fun. You have to have fun doing it too. It will snowball." (P_4)

##### Limiting factors

The limiting factors that were named were student clothing (*n* = 5/14), school structures (*n* = 4/14), storage of materials (*n* = 4/14), and weather (*n* = 4/14). Teachers found Service Center or school rules to be barriers of going outside. They also find that OL equipment requires large spaces for storage.

"One of the things also that is a problem sometimes is, I don't want to get too long, I’ll go fast, the School Service Center sometimes they can get in the way. It's often the big machines, it's often hard to get them to move, but when you're persistent, when you've proven yourself a little, when the school board or the service center sees that you're serious about what you're doing, you can open doors and get things changed." (P_1)

Finally, a preschool teacher pointed out the presence of many training opportunities, which however do not seem to be in line with her needs.

"That's it, but I find that there is like a great offer of training. But I don't know? Then maybe, you seem to have taken some good ones but it seems like there is too much training. Sometimes, I am doing it, yes, but I already do that. I went to the preschool training, I said, okay, but now I really want to go to a workshop where is it when I read the description, but I'm already doing it, I don't want help to start, yeah there's a lot of it, but it's how to start." (P_11)

### Emerging data

Additional findings emerged from the group interviews and participant observations that are worth mentioning. First, teachers named their motivations for incorporating OL into their practice. The reasons most given were (1) getting students outside (*n* = 6/14), (2) the COVID-19 pandemic (*n* = 5/14), (3) getting students to transfer learnings in OL to home (*n* = 3/14), and (4) fostering integration of immigrants (*n* = 2/14). Second, several teachers discussed the perceived positive effects of OL on their students. They named (1) feeling free (*n* = 7/14), (2) being more physically active (*n* = 5/14 and 2 observations), and (3) calming (*n* = 5/14 and one observation). Third, teachers named the perceived positive effects of OL for themselves as (1) stronger bonding with students (*n* = 4/14), (2) enjoyment (*n* = 4/14 and one observation), (3) feeling free (*n* = 3/14), and (4) two-way learning (*n* = 3/14). Teachers highlighted many benefits for their students and themselves.

## Discussion

The purpose of this study was to provide a recent portrait of the integration of OL in preschool and primary school teaching in Quebec. First, it appears that teachers’ perception of OL includes five major elements that are fairly unanimous (e.g., being outdoors and having the presence of nature). Secondly, teachers seem to aim for various pedagogical intentions in OL (e.g., environmental awareness, interdisciplinary learning), with different school subjects (e.g., French, mathematics) and learning activities (e.g., walking, doing ephemeral art) and in different settings (e.g., schoolyard, municipal park). Finally, various facilitating (e.g., parental support, volunteer support) and limiting factors (e.g., storage of materials, administrative structures), as well as teaching strategies (e.g., flexible planning, established routines), appear to influence teachers’ integration and organization of OL in the school setting.

### The outdoors as an educational approach

Teachers perceive OL as a pedagogical approach with five characteristics: (1) being outdoors, (2) having the presence of nature, (3) practicing a physical activity, (4) providing physical freedom, and (5) targeting a pedagogical intention. To the best of our knowledge, few studies in the scientific literature have focused on preschool and primary school teachers’ OL design. This research has therefore made it possible to better characterize it, at least for Quebec. Although the authors do not seem to be unanimous in their definition of OL (Auger et al., 2021), some highlight two central characteristics that are consistent with the results of this study: (1) the presence of a natural environment and (2) a certain amount of physical effort related to the activity practiced (Auger et al., 2021). The results of the present study also point to more specific characteristics of OL, such as being outdoors, providing physical freedom, and targeting a pedagogical intention related to an outdoors activity. This last characteristic, intention, seems to be part of a conception of outdoors that is specific to OL, through the intended learning intention. It seems, therefore, that for teachers, outdoors can take shape in different ways depending on the individual who uses it.

### Interdisciplinary intentions

We note that the majority of participating teachers seem to have several pedagogical intentions at the same time, such as aiming for more ownership of natural settings by students (e.g., discover the neighborhood), situating learning according to students’ emerging interests (e.g., learn the names of the birds you hear), and environmental awareness (e.g., learn the life cycle of a tree). The results indicate that teachers do not only seem to use outdoors as a setting context where learning and teaching take place (Legendre, 2005), but also as a pedagogical tool and as a lever for the integration of interdisciplinary learning (Moffet et al., 2019). Indeed, the participating teachers seem to conceive and use outdoors in an interdisciplinary way by crossing, varying, and connecting learning from different disciplines. In particular, basic school subjects such as mathematics or French seem to be part of an interrelated dynamic with environmental awareness or physical and health education content (e.g., work on a cartesian plane in a physical education course). Furthermore, beyond the educational opportunity, teachers seem to be well informed about the positive effects of outdoors for themselves and their students. OL appears to be implemented by several teachers to contribute favorably to children’s development and health, while optimizing their learning experience.

### Diversified and contextualized school subjects and learning tasks

This study identified various school subjects and learning tasks used in OL Regarding the most used school disciplines, this study supports data from the recent report by Ayotte-Beaudet et al. (2022), in which French, mathematics, physical and health education, and science are among the most taught areas in OL. In addition, this study identified learning activities specific to preschool and primary OL, such as walking, free play, building a shelter in nature, ephemeral art, running, lighting a fire, and cooking. We note that the school subjects and learning tasks used by the teachers are diversified and contextualized to outdoors. Some learning tasks also seem to have been transposed or adapted to the outdoor environment used (e.g., ephemeral art, cooking), or to be achievable only outdoors (e.g., building a shelter in nature). Furthermore, the teachers seem to go beyond the academic framework prescribed by the program and adapt their learning content according to their knowledge and skills in outdoors. A few preschool teachers also seemed to integrate spiritual and First Nation communities’ values in their outdoor practice, such as cooking bannock bread, setting up a native tent or performing a gratitude ritual.

### Accessible outdoor settings

In the present study, the school subjects and learning tasks used by teachers in OL were predominantly conducted in settings that were close to the school and therefore within walking distance. The settings most used by participating teachers were: (1) city parks, (2) school grounds, and (3) woodlands. These results are consistent with those of the research report on teaching practices in OL (Ayotte-Beaudet et al., 2022), which also names these three settings as the most accessible according to the teachers. Thus, we see that accessibility seems to play a major role in teachers’ use of the outdoor environment. Therefore, there seems to be a need to ensure that different outdoor settings are accessible within walking distance of schools in order to encourage their use in OL.

#### Association of results with the intentions to use the outdoors matrix

The results that emerged from the group interviews and participant observations allow for the association of the studied teaching practices with the intentions to use the outdoors matrix (Gadais et al., 2021a). First, all of the participating teachers are engaged *in* the outdoors, as they all take their classes outside (Gadais et al., 2021a). Similarly, a few teachers seem to draw on the *Udeskole* approach in their practice, as they implement mandatory and regular educational activities outside the school walls (Bentsen and Jensen, 2012), including walking around the surrounding neighborhood or visiting cultural venues or markets. These practices are carried out *via* and *for* the outdoor environment, as they appear to have an intention of awareness or reconnection to nature or the environment (Gadais et al., 2021a) and as they use nature for learning purposes. Second, the results indicate that outdoor activities are used by a majority of physical and health education teachers, such as snowshoeing, skiing, or skating, practiced in woodlands or city parks near the school. These activities are designed and intended to be practiced outdoors (Gadais et al., 2021a), such as rock climbing or kayaking, which are characterized by movement in an outdoor environment (Testevuide, 1996; Schnitzler and Saint Martin, 2021). Third, three preschool teachers have pedagogical intentions that are achieved *via* the outdoors, as they use nature as a means to a specific end or for the effects produced on students (Gadais et al., 2021a). In their practices, nature is used for learning purposes, exploring, discovering, or experimenting in a natural setting, such as making a shelter in the forest. Similarities are present between their practice and the *Forest School* approach, as they spend the majority of their days in the forest, in a variety of weather conditions, and encourage learning through free play and risk taking (Elliot et al., 2014; Coates and Pimlott-Wilson, 2019). Fourth and last, many teachers appear to be doing activities *for* the outdoor environment, as they have a pedagogical intention that is directly related to the environment (Gadais et al., 2021a), and thus aligns with the aims of *Environmental Education* (Sauvé, 2015). Through activities such as learning about the tree cycle, or developing the no-trace principle in outdoors, teachers aim for children to have a greater understanding, sensitivity, and connection to the environment.

Finally, the results of this study allow for an open dialogue about the intentions to use the outdoors matrix (Gadais et al., 2021a). First, it seems important to consider that the intentions to use the outdoors should not be considered exclusive to each of the spheres, as proposed by Gadais et al. (2021a), but should rather reflect the intentions of the teachers, which are often multiple. The different spheres of the matrix should thus intersect in order to allow the association of several intentions with a single task or activity. Secondly, we observe that many teachers use elements of nature (e.g.: leaves, branches, rocks, etc.) for learning purposes (e.g.: ephemeral art, discovery, etc.), without necessarily having an outdoor goal. These learnings often follow a logical progression, which does not seem to have been considered in the model of Gadais et al. (2021a). Furthermore, some of the activities presented by teachers, such as learning about the tree cycle, seem to be more in line with an intention *about* the environment. This proposed nuance between an activity having an intention *for* and *about* the outdoor environment would merit further investigation to determine if there are characteristics specific to each intention that can be supported by the scientific literature.

### Implicit and libertarian teaching

Findings from interviews and participant observations indicate that many preschool teachers appear to be many to use implicit pedagogy in their practice, that is, pedagogy that places the student in a situation of autonomy, without the teacher clearly integrating the learning content outdoors (Gauthier et al., 2013; Visioli, 2019). They do this through emergent pedagogy, which is a pedagogic style that encourage children to see themselves as the creators of their own learning (Dalke et al., 2007). In order to do this, teachers aim for student autonomy, by placing their interests at the heart of their learning process (Visioli, 2019). At the primary level, teachers seem more inclined to use explicit pedagogy with students, where the teacher acts as a guide in the development of their learning (Gauthier et al., 2013; Visioli, 2019).

Furthermore, the majority of participating teachers, both preschool and primary, appear to adopt a libertarian teaching style (Visioli, 2019) in OL. This teaching style seems to be reinforced when teachers are outside of the school perimeter, either in a wooded area or a nearby city park. The logbook supports this idea, believing that these settings would allow teachers to establish a framework, rather than total control, over the students’ learning process. Outdoor environments attended outside of the school perimeter would therefore allow for greater latitude in terms of student decision-making and autonomy (Visioli, 2019).

### Benevolent pedagogical relationship

Participant observations identified a caring and benevolent pedagogical relationship (Visioli, 2019) as well as a relational closeness between teachers (agents) and students (subjects) (Legendre, 2005). OL seems to bring hazards in relation to weather conditions and thus teachers seem to have to often deal with wellness-related issues in their students. This, therefore, seems to push them to engage in caring preventive behaviors before and during their activities. Teachers appear to be approachable, passionate, concerned about students and act as a model for students, four strategies mentioned by Pianta (1999) to foster the teaching relationship. Finally, several teachers also mentioned learning along with their students in OL, a position that would make them co-learners (Bergeron, 2020).

### Organized and flexible planning

The results of this study allow us to identify two qualities that are essential to optimal planning in OL, namely organization and flexibility. First, according to Yinger (1979), organization helps to simplify the teaching task. Our study reinforces this idea, since teachers indicate in a consensual manner that organization makes it possible to facilitate the unfolding of outdoor sessions, in addition to facilitating the management of unexpected events. Secondly, the majority of teachers mentioned the importance of flexibility in planning for OL. This flexibility, which they also seem to associate with a good capacity for adaptation, confirms the comments of Tochon (1993), who names the importance of adaptability relative to unpredictable factors in teaching. Finally, it is possible to observe a form of planned improvisation in the planning of the teachers, who seem to be experts and therefore have more than 8 years of experience in OL (Tochon, 1993).

### Group management with clear rules and a well-established routine

Teachers appear to adopt several effective instructional strategies to facilitate group management, specifically in OL. First, the use of clear, structured, and well-understood rules by students (Tessier et al., 2013) could be the source of effective group management in OL. For the teachers in this study, establishing rules related to geographic boundaries and student safety seemed to promote student understanding and task flow (Méard and Bertone, 2009). The use of clear and structured rules would therefore allow for a better internalization of the rules on the part of the students and would thus facilitate the conduct of the sessions outdoors. Second, planning and establishing routines would also be part of an effective group management approach in OL. Routines would increase the predictability of the course of action, flexibility and effectiveness of teaching (Yinger, 1979; Tochon, 1993), factors that seem important to consider in OL. To this end, Méard and Bertone (2009) assert that student understanding and task flow are optimized by repeating prescribed rules. The results of this study confirm that, when well planned and integrated, routines seem to ensure that teaching-learning outdoors goes smoothly.

### Facilitating factors: Human, material and financial support

This study identified factors that facilitate the use of the OL in preschool and primary settings that had already been identified by the literature, such as (1) support from the educational community for outdoor integration (school team/management/community) (Maziade et al., 2018) and (2) material and financial support (Maziade et al., 2018; Sport et loisir de l'Île de Montréal, 2019). New factors that would facilitate the use of the OL in preschool and primary settings also emerged from this research. Specifically, these included (1) parental support and awareness about OL and (2) volunteer support. Finally, some teachers even indicated that one should not hesitate to go for it, to dare and that fun is part of the recipe in order to share rich and authentic moments with the students in OL.

### Limiting factors: Existing structures

Among the limitations that emerged in this study and that confirm those already listed, we find (1) the lack of funding (Waite, 2010; Sport et loisir de l'Île de Montréal, 2019), (2) the lack of time to prepare and carry out activities (Edwards-Jones et al., 2018; Sport et loisir de l'Île de Montréal, 2019; Van Dijk-Wesselius et al., 2020), and (3) weather conditions (Ruether, 2018). The lack of support in learning outdoors (Ruether, 2018; Sport et loisir de l'Île de Montréal, 2019) was qualified by a preschool teacher as a lack of fit between the training offered and the needs of teachers. The lack of material and human resources (Sport et loisir de l'Île de Montréal, 2019) was rather qualified in this study by the lack of volunteer presence and the lack of support for student clothing. Moreover, this study brought to light other limitations among teachers that, to our knowledge, have not been mentioned in the literature: (6) storage of materials and (7) administrative structures. By storage of materials, we mean the space to store equipment related to outdoor activities, which seems to be insufficient for several teachers. By administrative structures, we mean all the administrative procedures and rules in place in schools and Quebec School Services Center with which teachers are often confronted with in their OL practice. Finally, several limitations to the integration of the outdoors in the school setting were named by the participants in this study, but the teachers consensually demonstrated that they were not unavoidable, since they seemed to find workaround solutions.

### Strengths and limitations of this study

To our knowledge, this study is the first in Quebec to take such a detailed look at the teaching strategies present in OL and among preschool and primary school teachers. Moreover, the methodological triangulation allows for greater credibility of the research results, which were studied by two different and complementary instruments, namely group interviews and participant observation. Finally, the sample included participants from several regions of Quebec, which allowed for the diversification of the fields of practice outdoors and for a variety of results, in terms of context, pedagogical intentions and settings.

This research project also has certain limitations. First, data collection was primarily conducted during the winter months. Further studies of teaching practices in the fall and spring are needed to provide a more complete picture. This would provide more complexity in characterizing the uses and teaching strategies used in OL. Second, specialist teachers who use outdoors, such as in drama or music, were not included in this study. Studying the teaching practices of all specialist teachers would allow for a greater breadth of results. Third, since some interviews were conducted prior to the observations, there is a possible influence between the two datasets. Therefore, only the primary researcher participated in the data collection and the co-authors served as referees to ensure a more neutral posture in the data analysis.

## Conclusion

In conclusion, the purpose of this study was to portray the integration of the outdoors in Quebec in preschool and primary school education by exploring the perception, uses, teaching strategies and influencing factors present in OL.

### Main results

The results of this study have allowed new knowledge to emerge with regard to OL in Quebec. First, it revealed the teachers’ perception of the outdoors that includes five main elements: (1) being outdoors, (2) having the presence of nature, (3) practicing a physical activity, (4) providing physical freedom, and (5) targeting a pedagogical intention. Second, the results indicate that the uses of the OL in schools are varied, both in terms of school subjects and learning tasks (e.g., French, walking), pedagogical intentions (e.g., environmental awareness, interdisciplinary learning), and in terms of the frequented environments (e.g., woodlands, municipal parks). Third, certain teaching strategies were identified by this study to facilitate teaching outdoors (e.g., structured and flexible planning). We believe that these teaching strategies will have an important contribution for OL practices, as they fill a scientific gap, particularly in terms of group management (Ayotte-Beaudet et al., 2022) and pedagogical tools (Maziade et al., 2018). Finally, the results of this study have brought to light new factors that help (e.g., parental support) or limit (e.g., administrative structures) the integration of the outdoors by teachers and that are complementary to those already raised by the scientific literature.

### Future perspectives

In order to recognize the outdoors as a pedagogical tool or as a lever for education in Quebec, it is important to emphasize the diversity of contexts and possible intentions in OL. This study has brought to light new avenues for promoting OL in Quebec, which would benefit from expansion in schools. To echo the reflections of Maziade et al. (2018), we consider that the Quebec Education Program (PFEQ – *Programme de formation de l’école québécoise*) does not currently include the outdoors sufficiently in its pedagogical content, despite the advice issued by the Ministry of Education to promote the inclusion of the outdoors in schools (Ministère de l’Éducation et de l’Enseignement supérieur du Québec, 2017). The burden of administrative structures for OL in teaching could be alleviated with greater inclusion and recognition of its use in the PFEQ. Finally, approaches that have aims *for* or *about* the outdoors should be further studied, as they appear to have significant educational potential in terms of environmental awareness and the development of conscious and engaged eco-citizenship in children (Sauvé, 2015). It is crucial to look into them more seriously in order to develop their critical thinking towards environmental issues and to generate responsibility and proactivity for the environment, especially in response to the climate emergency we are currently experiencing (Agundez-Rodriguez and Sauvé, 2022). Finally, it seems fundamental for scientific research to take a more serious look at the potential of OL as societal change (Smith, 2002; Giroux, 2006; Glassner and Eran-Zoran, 2016; Agundez-Rodriguez and Sauvé, 2022).

## Data availability statement

The raw, anonymized data supporting the conclusions of this article will be made available by the authors upon request, without undue reservation.

## Ethics statement

The studies involving human participants were reviewed and approved by Comité d’éthique de la recherche pour les projets étudiants impliquants des êtres humains (CERPE - plurifacultaire). Written informed consent was obtained from the minor(s)’ legal guardian/next of kin for the publication of any potentially identifiable images or data included in this article.

## Author contributions

A-AB and TG were involved in the design of the study and contributed to the review of literature. A-AB conducted analyses and wrote the results section. A-AB wrote the first draft of the manuscript, after which YL, CK, and TG read and contributed to the revision of the manuscript. All authors contributed to the article and approved the submitted version.

## Conflict of interest

The authors declare that the research was conducted in the absence of any commercial or financial relationships that could be construed as a potential conflict of interest.

## Publisher’s note

All claims expressed in this article are solely those of the authors and do not necessarily represent those of their affiliated organizations, or those of the publisher, the editors and the reviewers. Any product that may be evaluated in this article, or claim that may be made by its manufacturer, is not guaranteed or endorsed by the publisher.
