# Supplementary material for: When are you taking us outside? An exploratory study of the integration of the outdoor learning in preschool and primary education in Quebec
Source: Front Psychol. 2022 Oct 20;13:955549. doi: 10.3389/fpsyg.2022.955549 (PMC9631936; doi:10.3389/fpsyg.2022.955549)
Supplement: Supplementary file 1 [file Table_1.PDF]

Additional file. Focus group interview guide

Introduction

a) Instructions about the research project and the focus group;
b) Filled and signed consent form;
c) Focus group's instructions and guidelines.

Semi-structured focus group, talk time assessed by the principal investigator, maximum 90 minutes. Give concrete examples, if possible.

Themes	Sub-themes
Introduction	Could you discuss about the outdoors activities you provide to your students?<br><br>Where are your outdoor classes with your students taking place?<br><br>Since when have you integrated outdoors into your teaching practice?
Perception of the outdoors	What does outdoors mean to you?<br><br>What motivates you/what are the reasons that are pushing you to integrate outdoors into your practice?<br><br>• According to you, what elements would bring a teacher to use outdoors into his or her teaching practice?
Uses of OL	Could you describe the top three outdoors environments that you visited the most with your students during the year?<br><br>• According to seasons (fall/winter/spring)<br>• According to its proximity or farness<br><br>What are the school subjects, the means of action, the learning tasks or the types of activities that you offer to your students during an outdoors class?<br><br>• During the last year<br>• According to seasons (fall/winter/spring)
Teaching strategies and factors that influence OL	What are the facilitators and the barriers to the integration of outdoors into the teaching practice in your school?<br><br>• As a teacher?<br>• Into the school team ?<br>• From an administrative point of view?<br><br>What are the stakes you encounter when you bring your students outside? (e.g., dressing up, gear, rules)<br><br>• Fall/winter/spring

	What organization routines do you use when you teach inside? Outside?<br><br>• Differences and similarities<br><br>What are the main strengths of a planification/preparation of a class given outside?
Conclusion	Additional comments or themes not covered that you would like to discuss?<br><br>Thank participants for their time
